# The Outcome of Trans-catheter Closure of Patent Ductus Arteriosus: A Single-Center Experience

**DOI:** 10.7759/cureus.21577

**Published:** 2022-01-24

**Authors:** Abdul Moeed Khan, Zia Ullah, Saadia Ilyas, Haseen Dil Wazir, Yasir Rehman, Ijaz Hussain, Haleema Sadia

**Affiliations:** 1 Pediatric Cardiology, Peshawar Institute of Cardiology, Peshawar, PAK; 2 Pediatric Cardiology, Hayatabad Medical Complex Peshawar, Peshawar, PAK; 3 Pediatric Cardiology, Lady Reading Hospital, Peshawar, PAK; 4 Pediatric Cardiology, Peshawar institute of Cardiology, Peshawar, PAK; 5 Medicine, Jinnah Medical Institute, Peshawar, PAK

**Keywords:** outcome, embolization, device closure, trans-catheter, pda

## Abstract

Objective

The objective is to share our experience of trans-catheter device closure of patent ductus arteriosus (PDA) and review its efficacy and immediate complications.

Methodology

This cross-sectional observational study was done in the Department of Pediatric cardiology, Lady Reading Hospital, Peshawar from January 2020 to December 2020. A total of 51 patients with isolated PDA, who underwent PDA device closure, were enlisted in this study through consecutive sampling methods, irrespective of their age and gender. Data of the patients were collected on preformed pro forma. Data was entered in SPSS 20 (SPSS Inc., Chicago, IL, USA). Descriptive statistics like mean ± SD for numerical data (age, weight, and hospital stay), while frequency and percentage for categorical variables (Device type, complication, hospital stay, and type of anesthesia given) were calculated.

Results

Among total 51 patients (33 [64.7%] females and 18 [35.3%] males) with mean age 8.19 ± 6.96 years underwent attempted trans-catheter PDA device closure. In all cases PDA was successfully occluded with appropriate size devices. General anesthesia was given in 38 (74.5%) patients. Complications occurred in only four (7.8%) patients in the form of hemolysis followed by acute kidney injury in one patient, another had device embolization to descending aorta, which was retrieved in cath lab, one had device embolization to the right pulmonary artery, which was retrieved in cath lab and one had mild left ventricular systolic dysfunction. The mean duration of hospital stay was 22.0 ± 10.2 h.

Conclusion

Trans-catheter PDA device closure is a safe procedure with a high success rate and little morbidity.

## Introduction

Patent ductus arteriosus (PDA) is a vascular communication between the left pulmonary artery with the descending aorta just after the origin of the left subclavian artery. It is an essential fetal structure that closes spontaneously in about 90% of infants during the first 48 hours of life. Persistent patency of the ductus arteriosus beyond a few weeks is considered abnormal [1]. PDA constitutes 6%-11 % of all congenital defects and occurs in about one in 2,500-5,000 live births. Significant sized PDA causes symptoms of heart failure and poor weight gain in children [2,3]. PDA closure is recommended in every patient who presents with left ventricular volume overload even with no symptoms and no pulmonary artery hypertension (PAH) and device closure is the treatment of choice when technically possible [4]. The treatment of clinically silent or small PDAs is debatable [5].

The first surgical closure of PDA was done by Gross and Hubbard in 1939 [6] and the trans-catheter PDA closure was later done by Portsmann et al. in 1967 after that there has been significant progress in the devices used for closure of a PDA [7].

Although the surgical approach is very efficacious and with very low mortality it is associated with greater morbidity (like pneumothorax, bleeding, recurrent laryngeal nerve injury), greater postoperative pain, and longer hospital stays than trans-catheter techniques. These factors have been the reasons that percutaneous PDA closure has rapidly become the first choice for PDA closure in patients with suitable anatomy [1].

The risks (including device embolization, residual leak, thromboembolism, cardiac perforations, and vascular injuries) associated with PDA device closure are minimal and also depend on the center as well as the operator [8]. In Pakistan, limited studies regarding the complications and outcomes of trans-catheter approach have been published. Studies have reported mortality in 0.2% and significant complications in 3.8% [8,9].

## Materials and methods

Study design

This descriptive cross-sectional study was conducted at Lady Reading Hospital Peshawar, Pakistan. Data collection was started in January 2020 and completed in December 2020.

Inclusion and exclusion criteria

Patients with isolated PDA irrespective of age and gender were included in the study. Patients of weight less than 8 kg, other associated congenital lesions, significant pulmonary hypertension, and syndromic features were excluded.

Data collection

Fifty-one patients were enrolled with consecutive sampling techniques. All patients underwent detailed pre-procedural assessment including detailed history & physical examination, electrocardiography, Chest x-ray, full blood count, and detailed trans-thoracic echocardiography. 2D & Color Doppler was used to determine the size of the PDA. The diameter at the narrowest point of a ductus was then assessed on a frozen image. Left ventricular ejection fraction was measured in all patients using M-Mode echocardiogram. All cases were performed by a Consultant Pediatric cardiologist. After taking informed consent, patients were shifted to the catheterization lab. Either general or local anesthesia was given depending upon age and level of cooperation. Sheaths were passed in both the right femoral vein and artery. A pigtail catheter was used for an aortogram which was performed in full lateral view to determine the size, shape, and narrowest diameter of the PDA. Multipurpose catheter A2 was advanced through the PDA from the pulmonary side into the descending aorta and was then exchanged with a delivery sheath, over a 0.035" exchange length 260cm wire. The appropriate device was passed through the delivery sheath into the descending aorta and the aortic end of the device was deployed. The sheath and the device were pulled back as a single unit, and the rest of the device was then uncovered within the duct. Post-device deployment aortogram was performed to confirm device position and to evaluate residual leak. The device was released after acceptable positioning was ascertained. Post-procedure vitals signs, wound site along distal pulses were monitored. Post-procedure echocardiography was done before discharge and all patients were then followed for the next three months.

Statistical analysis

Data were entered in SPSS 20 (SPSS Inc., Chicago, IL, USA). Descriptive statistics like mean ± SD for numerical data (age, weight, and hospital stay), while frequency and percentage for categorical variables (complication and type of anesthesia given) were calculated.

Ethical considerations

This study was approved by the Institutional Review Board of Lady Reading Hospital, Peshawar, Pakistan, with the approval number 270/LRH/MTI.

## Results

In our study, a total of 51 patients were included. There were 18(35.3%) males and 33(64.7%) females. The age ranged from one year to 37 years and the mean of 8.19 ± 6.96 years. 38(74.5%) patients were in the age range <10 years, 10 (19.6%) patients were between 11 and 20 years old, two (3.9%) patients were between 21 and 30 years, and only one (2.0%) patient was >30 years as shown in Table 1. Out of 51, 38 (74.5%) cases were done under general anesthesia and 13 (25.5%) cases were done with local anesthesia. The mean duration of the hospital stay was 22.0 ±10.2 hours. In 48 (94.1%) patients, hospital stay duration was less than 24 hours; in two (3.9%) patients, it was between 48 and 2h and only one (2.0%) patient stayed >72 hours (Table 2). Echocardiography done before discharge confirmed all devices to be in place. Complications occurred in four (7.8%) patients. One patient developed acute hemolysis with a drop in hemoglobin and increased reticulocyte count followed by acute kidney injury. Patient vitals, input/output record, hemoglobin, urea, and creatinine levels were monitored throughout the stay. The stay in the hospital was 13 days. The patient was discharged after improvement. One patient had device embolization to descending aorta on one week follow up, which was retrieved in the cath lab. One patient had device embolization to right pulmonary artery in the cath lab after device deployment, which was retrieved by snaring in the cath lab. One patient developed mild left ventricular systolic dysfunction which improved on follow-up echo (Table 3).

**Table 1:** Hospital stay and type of anesthesia

		Frequency (%)
Hospital Stay	Mean ± SD	22.0 ± 10.2 h
	<24 h	48 (94.1%)
	48-72 h	2 (3.9%)
	>72 h	1 (2%)
Anesthesia	General Anesthesia	38 (74.5%)
	Local Anesthesia	13 (25.5%)

 

**Table 2:** Distribution of patients according to age, gender, and weight

		Frequency (%)
Age	Mean ± SD	8.19 ± 6.96
	<10 years	38 (74.5%)
	11-20 years	10 (19.6%)
	21-30 years	2 (3.9%)
	31-40 years	1 (2.0%)
Gender	Female	33 (64.7%)
	Male	18 (35.3%)
Weight	Mean ± SD	22.68 ± 14.27 kg

**Table 3:** Frequency distribution of complications

Complications	Frequency (%)
Device embolized to descending aorta	1 (2.0%)
Hemolysis leading to acute kidney injury	1 (2.0%)
Mild LV dysfunction (ejection fraction) EF 50%	1 (2.0%)
Device embolization to right pulmonary artery	1 (2.0%)
None	47 (92.2%)

## Discussion

PDA is a common congenital heart disease. It has a broad spectrum of clinical manifestations, varying from asymptomatic heart murmur to congestive heart failure or Eisenmenger’s syndrome. Closure of PDA via trans-catheter route has a very high success rate (97%-99%) [10]. The high success rate depends on the PDA anatomy and appropriate device size selection. In our study, PDA device closure was attempted in 51 consecutive cases over one year period with a 100% success rate. Brunetti et al. [11] reported successful closure of PDA in 357 out of 359 attempted device closures. Another study by Sultan et al. [8] also reported a high success rate of 98.2%. Most of the cases in our study were younger than 10 years (74.5%) and female patients (64.7%) outnumbered the males which is expected, as PDA is more common in the female gender as reported in most studies [12].

In this study, out of 51 cases, various PDA occluder devices were used to occlude the PDA. Occlutech 8/10 PDA occluder was used the most in 14 cases followed by Occlutech 6/8 PDA occluder in 11 patients. The operator preferred the use of Occlutech devices in most cases due to its low cost and easy availability in the regional market as well as high safety profile [13]. General anesthesia was given in 38 (74.5%) cases due to a higher number of younger children.

We encountered complications in four cases. There was one case in which the device (Occlutech 8/10 duct occluder) dislodged after its deployment. It was retrieved from the right pulmonary artery with a snare and redeployed in the cath laboratory. Another patient had embolization of the device to descending aorta. The patient had 12mm PDA with moderate pulmonary hypertension. After initial successful PDA closure with Occlutech 14/18 duct occluder, the patient was discharged but presented one week later with lower limb claudication. The device was retrieved and PDA closed with ASD device. The likely reasons for these dislodgments were primarily the choice of smaller occluders in view of the small ampulla and higher pulmonary artery pressures. In one study from Saudi Arabia, device dislodgment to a pulmonary artery occurred in six (2.9%) patients out of 205 cases. Out of these six cases, four patients needed surgical intervention [14]. Device dislodgment was noted in 3.9% of cases in our study which is similar to those reported by Khan et al. [14]. Noting the significant device embolization numbers, we strongly recommend that PDA device closure should be undertaken in a setup where device retrieval equipment and Pediatric cardiac surgery facilities are available. One patient who had normal left ventricular systolic functions (EF 65%) developed mild left dysfunction (EF 50%) with no clinical symptoms. This patient was followed for a period of three months post-procedure and the heart functions returned within the normal range on subsequent echo. Another patient developed hemolysis followed by acute kidney injury. In this patient, a relatively larger device Occlutech 14/18 duct occluder was used. The patient recovered with the use of steroids, hydration, and vigorous monitoring of vitals and output. Hemolysis can occur with or without residual shunts following PDA device closure. Amoozgar et al. [15] reported four cases of hemolysis out of total 138 cases following device closure of left to right shunts. Mortality of surgical PDA ligation has been reported to be as high as 2% in one study [16] while mortality from trans-catheter PDA closure is low. Sultan et al. [8] reported one (0.2%) case of mortality out of 491 cases of trans-catheter PDA closure. In our study, there was no mortality. 

The mean hospital stay after PDA device closure in a local study by Zulqarnain et al. [17] was 37.9 hours compared to 22 hours reported in our study. The results of our study are comparable to that of modern centers where it is reported to be <24 hours [18]. This study was limited to single-center with a moderate number of cases. To establish the efficacy and safety of PDA device closure in our region more studies with larger sample sizes from multiple centers need to be published.

## Conclusions

PDA is a common congenital heart defect, which if not closed leads to many cardiac and pulmonary complications. After the introduction of trans-catheter closure of PDA, it has become standard of care in closing most of PDAs. Our study also showed that trans-catheter PDA device closure is a safe procedure with a high success rate and with the advantage of little morbidity.
